# Comparison of three different treatment methods for traumatic and Iatrogenic peripheral artery pseudoaneurysms

**DOI:** 10.1111/os.13315

**Published:** 2022-06-09

**Authors:** Baocheng Zhao, Jinli Zhang, Jianxiong Ma, Mei Huang, Jin Li, Xinlong Ma

**Affiliations:** ^1^ Department of Orthopaedics Tianjin Medical University General Hospital Tianjin China; ^2^ Department of Traumatic Orthopedics Tianjin Hospital Tianjin China; ^3^ Biomechanics Research Department Biomechanics Research Institute Affiliated to Tianjin Hospital, Tianjin Hospital Tianjin China

**Keywords:** Endovascular intervention, Iatrogenic injury, Open surgery, Peripheral pseudoaneurysm, Ultrasound‐guided thrombin injection

## Abstract

**Objective:**

To compare the efficacy of open surgery (OS), endovascular interventions (EIs), and ultrasound‐guided thrombin injection (UGTI) for the treatment of peripheral arterial pseudoaneurysms (PAs).

**Methods:**

From January 1, 2001, to February 10, 2021, 38 patients diagnosed with traumatic and iatrogenic PAs treated with OS, EI, and UGTI were retrospectively analyzed. There were 18 females and 20 males, with an age of 56.47 ± 14.08 years (range,17–87 years). Anesthesia modality, operation duration, blood transfusion, duration of hospital stay, primary and secondary success rates, and complication rate were used to evaluate the surgical outcomes.

**Results:**

There were 11 cases under regional anesthesia and 4 under general anesthesia in OS group, 9 under regional anesthesia and 1 under general anesthesia in EI group, and no regional or general anesthesia was required in UGTI group. There was no significant differences between any two groups (*χ* ^*2*^ *=* 39.80, *p* < 0.05). The blood tranfusion amount (units) were 3.6 ± 6.0, 0.8 ± 2.5, 0.0 ± 0.0 for OS, EI, and UGTI groups, respectively, with significant difference between OS and UGTI groups (*F* = 3.03, *p* < 0.05). The operation duration (minutes) of OS, EI, and UGTI groups were 80.0 ± 41.9, 56.0 ± 8.4, and 22.7 ± 5.3, respectively, with significant difference between any two groups (*F* = 15.69, *p* < 0.05). The duration of hospital stay (days) were 47.7 ± 39.0, 31.5 ± 17.6, and 16.3 ± 9.5, repectively, with significant difference between any two groups (*F* = 47.73, *p* < 0.05). The primary clinical success rates were 80% (12/15), 90% (9/10), and 92.3% (12/13) in OS,EI, and UGTI groups, respectively, with no significant difference between any two groups (*χ* ^*2*^ = 0.34, *p* > 0.05). The secondary clinical success rates were 100% for all three groups. The overall complication rates of OS, EI, and UGTI groups were 20% (3/15), 10% (1/10), and 7.7% (1/13), respectively, with no significant difference between any two groups (*χ* ^*2*^ = 1.00, *p* > 0.05). The infection rates were 13.3% (2/15), 10% (1/10), and 0% (0/13) in OS, EI, and UGTI groups respectively, with no significant difference between any two groups (*χ* ^*2*^ = 1.80, *p* > 0.05). The reintervention rates were 6.7% (1/15), 0% (0/10), 7.7% (1/13) in OS, EI, and UGTI groups, respectively, with no significant difference between two groups (*χ* ^*2*^ = 0.95, *p* > 0.05). Neuralgia was relieved in all patients.

**Conclusions:**

OS, EI, and UGTI are efficacious and safe options for the treatment of appropriate patients with traumatic and iatrogenic PAs. UGTI would be considered as a first‐line therapy for this condotion.

## Graphical abstract

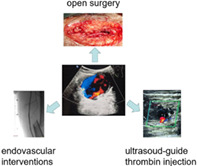

The open surgery, endovascular intervention, and UTGI are efficacious and safe for treatment of appropriate patients with peripheral artery PA. The patients should be treated individually.

## Introduction

Peripheral arterial pseudoaneurysm (PA) is particularly rare, with a reported incidence of 0.06% to 7.7%^1^, ^2^. The majority of PAs were secondary to trauma (penetrating or blunt injury) and iatrogenic injury^3^. The pathogenesis of PA remains unclear^4^. Delayed diagnosis and treatment may result in severe complications, such as peripheral nerve compression, distal extremity ischemia, thromboembolism, and even catastrophic rupture and hemorrhage^5^, ^6^, ^7^. Therefore, timely diagnosis and proper management are of great clinical significance.

Treatment options vary from conservative treatment to open surgery (OS), and in recent years, to minimally invasive interventions such as endovascular intervention (EI) and ultrasound‐guided thrombin injection (UGTI)^3^, ^5^, ^6^, ^8^. The choice is determined by the location, size, symptoms, and donor artery^8^. Recent surgery results are satisfactory, leading to the consideration of these surgical procedures as efficacious therapies^3^, ^9^, ^10^. Nevertheless, all treatment methods have their own merits and drawbacks.

OS is an important treatment option for PAs with the advantage of a high rate of long‐term vascular patency and the disadvantages of major blood loss and infection^2^. Indications for open surgical repair of PAs include failed minimally invasive vascular intervention, rupture or imminent rupture, infections, associated arteriovenous fistula, a location above the inguinal ligament, neuralgia caused by nerve compression, and severe distal ischemia^3^, ^4^. Devendra *et al*. reported on 14 PA cases treated with OS following orthopedic trauma and recognized this treatment option as the standard therapy for PA^10^.

Although OS is considered the standard of care, treatment for PA has evolved in recent years from OS to minimally invasive interventions, such as UGTI and EI. Minimally invasive interventions have the advantages of decreased morbidity and mortality rates. UGTI has a high success rate of more than 90% and a low complication rate^6^, ^11^. This treatment is effective in achieving thrombosis of the PA sac. UGTI is widely used for PAs with its indications extended to PAs of both upper and lower limbs^12^.

EI is an option for treating PAs with varying success rates. Gratl *et al*. reported that iatrogenic crural PAs treated with intraluminal stent grafting had poor long‐term patency^13^. Conversely, Criado *et al*. reported all covered stents remained patent at 2–19 months of follow‐up in five PA patients treated with endovascular repair^14^. EI may be reserved as an alternative method in selected cases. This technique may be inferior to UGTI and OS in both efficacy and cost^2^, ^15^. EI is utilized more often for arteriovenous fistulas, emergent ruptures, and failed compression and/or thrombin injection, as well as for PAs that are difficult to access percutaneously or have a high potential risk of major morbidity and mortality if managed by OS^2^, ^15^. In spite of its advantages, the incidence of lumen thromboembolism should still be considered^2^. EI is optimal for arteries with relatively large diameters, due to a higher risk of thrombosis in small arteries. Because of the small sizes of the brachial, radial, and ulnar arteries, the use of endovascular techniques is limited in the upper extremities. However, case series of EI at such locations have been reported in the literature^16^.

To date, there is still controversy over the optimal treatment for PA^2^, ^8^, ^9^, ^10^. Although OS is considered the standard treatment method with good results, some authors report that UGTI is safer and more effective than OS for treating selected groups of PA patients^10^, ^11^. To our knowledge, there is no research comparing OS, EI, and UGTI as treatment of PA. Due to the small number of case series reported in most circumstances and the absence of randomized clinical trials, no standard treatment protocol has been proposed.

Taking this into consideration, we retrospectively analyzed the data of OS, EI, and UGTI for PA treatment in our hospital. This study aims to: (i) compare the efficacy and safety of the three aforementioned methods for treating traumatic and iatrogenic PA; (ii) discuss the choice of operation; and (iii) attempt to propose a pragmatic treatment strategy.

## Materials and Methods

The study was approved by the Clinical Research Ethical Committee of Tianjin Hospital, with an approval number of 2022‐076. From January 1, 2001, to February 10, 2021, 49 patients diagnosed with traumatic and iatrogenic PA undergoing OS, EI, and UGTI were identified. A total of 38 patients were enrolled in the study, according to the inclusion and exclusion criteria. The patients were divided into three groups based on the treatment modality: OS, EI, and UGTI.

The inclusion criteria are as follows: (i) age > 15 years; (ii) PA caused by trauma or iatrogenic injury; (iii) diameter of sac >2 cm; and (iv) treated with UGTI, EI, or OS.

The exclusion criteria are as follows: (i) asymptomatic patients; (ii) PA sites at the wrist, hand, foot, or ankle: (iii) infected PA: (iv) ruptured PA; and (v) accompanying arteriovenous fistula.

### Imaging modality

Duplex ultrasonography was used as the first‐line imaging modality for screening and surveillance. This technique can delineate the size of PA, the thrombus within the sac, and shows the patency of the donor arteries. On B‐mode imaging, PA shows an echolucent sac adjacent to the artery. The color Doppler may demonstrate a characteristic “yin‐yang sign” of a whirling flow pattern. The diagnosis relies on the image of sac's neck and “to‐and‐for” waveform between the donating artery and the sac, indicating flow in and out of the pseudoaneurysm sac.

### Sac size measurement

Each PA was assumed to be an ellipsoid and the volume was estimated by the formula:

$$\text{Volume}=4/3\times \pi \;\left(\text{length}/2\right)\times \left(\text{width}/2\right)\times \left(\text{height}/2\right).$$

The mean size of the PAs was 160 cm^3^ (range, 0.96–5670 cm^3^).

### Treatment workflow

**Fig. 1:**
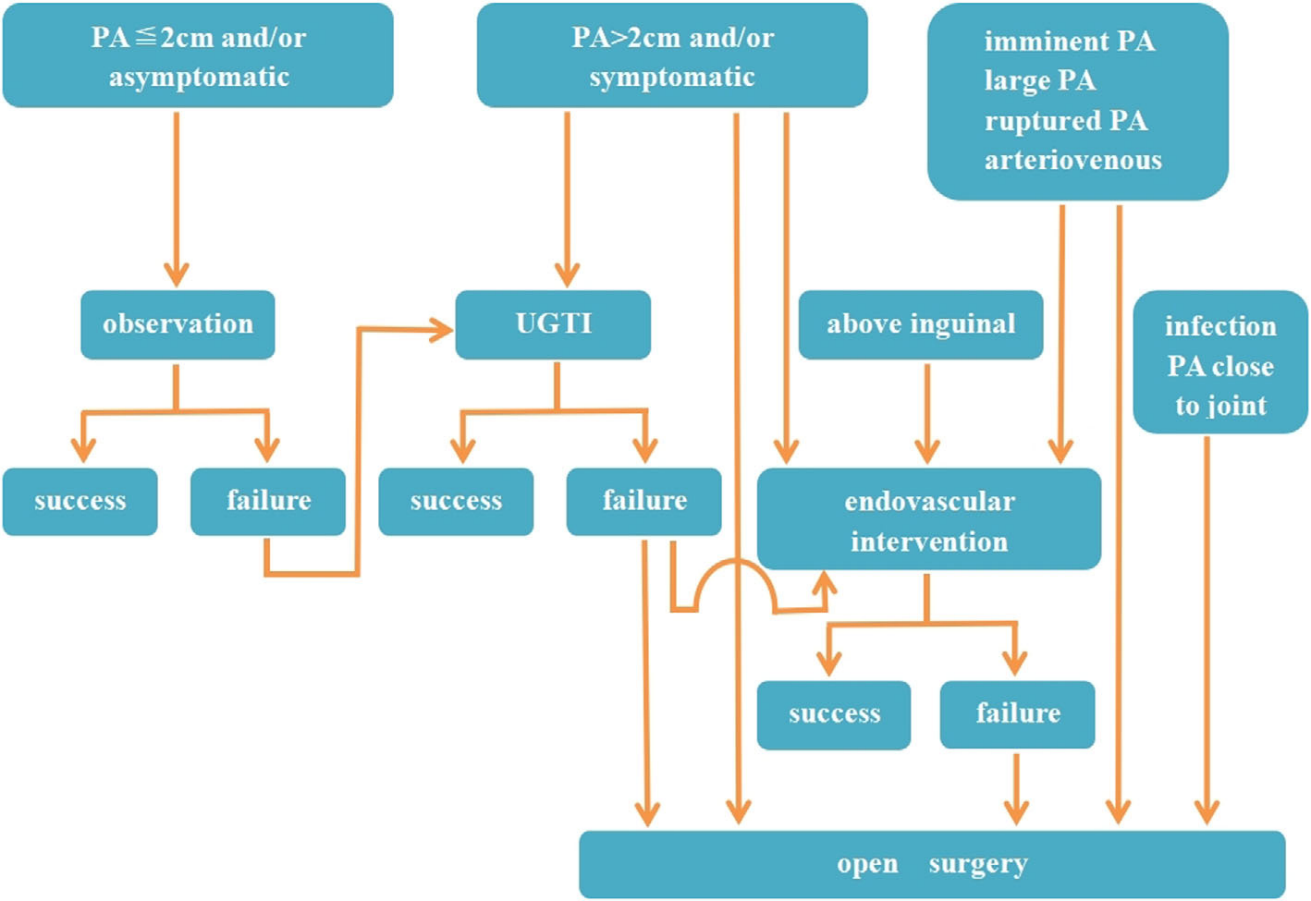
Flowchart of treatment options for PA

Our workflow of treating PA patients is shown in Fig. 1. Patients with asymptomatic PA with diameter ≦2 cm are observed for spontaneous thrombosis. For patients who failed in spontaneous thrombosis, or those with diameter >2 cm, UGTI is undertaken as a first‐line option. If UGTI fails despite two attempts within 48 h, EI or OS is then recommended. In most circumstances, OS is reserved for patients with complicated PA, and those who failed with UGTI and/or EI.

#### Surgical procedures

All of the surgeries were performed by senior doctors: J.L. Zhang, M. Huang, and J. Li independently.

#### Open surgery ( *OS* )

**Fig. 2:**
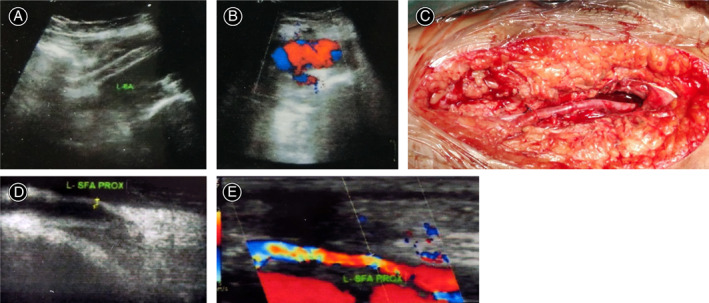
A 17‐year‐old patient with a dilated and pulsatile mass in the ilioinguinal fossa. (A, B) Duplex sonography reveals the PA. (C) The common femoral artery is repaired with autogenous saphenous vein graft interposition and sac excision. (D, E) Duplex sonography demonstrates a patent common femoral artery 4 months after operation

OS was performed under regional or general anesthesia. The surgical option was decided individually. Excision with arterial ligation was indicated for expendable minor arteries with good collateral circulation and no distal ischemia. Excision with end‐to‐end anastomosis was performed for vital arteries with a defect size ≦2 cm, while excision with lateral repair was performed for minor injuries of local artery walls. Excision with autogenous saphenous vein graft interposition was preferred for vital arteries with a defect size >2 cm (Fig. 2).

#### Endovascular Interventions ( *EIs* )

**Fig. 3:**
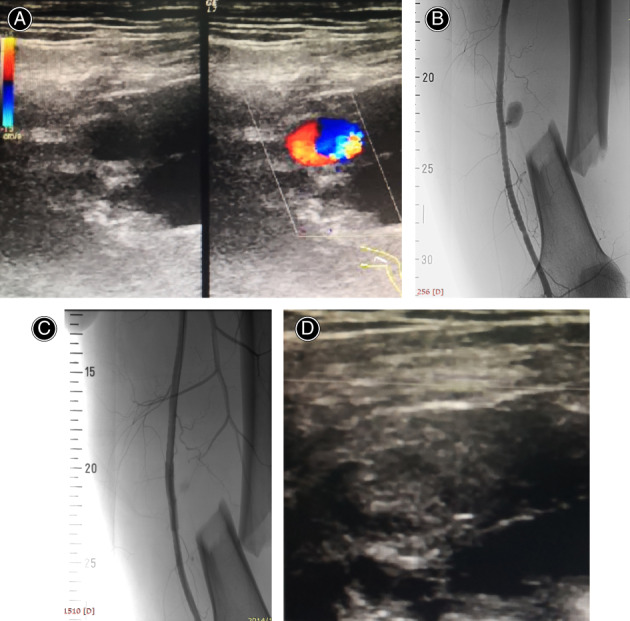
A 44‐year‐old patient with superficial femoral PA following femoral fracture at the junction of the middle and lower thirds. (A) Arterial Duplex sonography demonstrates a superficial femoral PA. (B) Angiography shows superficial femoral PA with active extravasation of contrast. (C) PA is excluded by covered stent grafting and normal blood flow in the superficial femoral artery is observed. (D) Color Duplex sonography shows normal blood flow in the superficial femoral artery with no “to‐and‐fro” blood flow in the PA sac

EI with covered stent graft/coil was performed in a digital subtraction angiography suite under regional or general anesthesia^14^. The contralateral femoral artery in the lower limbs or contralateral brachial artery in the upper limbs was typically used as the site for access *via* the Sieldinger technique. After the procedure, an angiogram revealed stasis of blood flow that resulted from selective embolization or stenting (Fig. 3).

#### Ultrasound‐Guide Thrombin Injection ( *UGTI* )

**Fig. 4:**
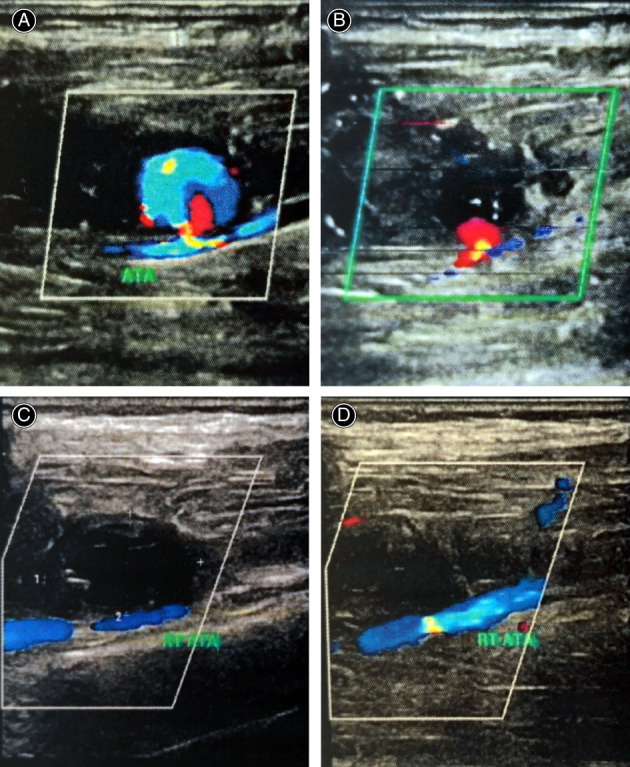
A 43‐year‐old patient with a mass on the lower leg. (A) Color Duplex sonography demonstrates an anterior tibial PA. (B) Color Duplex sonography shows thromboembolization in the sac during UGTI. (C, D) Color Duplex sonography shows the thrombosed PA with no blood circulation signal in the sac after operation

UGTI was performed as described by Kang *et al*.^12^ Under the guidance of ultrasound, a bovine thrombin solution (200–500 U/mL) of 1 to 5 mL was injected gradually into the sac. Thrombosis in the sac was monitored by ultrasonography. Distal pulses were assessed before and after the procedure. The ankle‐brachial index was measured after the procedure to confirm that no arterial embolization occurred (Fig. 4). Anticoagulation therapy was given to the patients routinely, and antiplatelet therapy to those who underwent minimally invasive interventions.

### Outcome measurements

The parameters for outcome evaluation comprised two aspects. First, procedural parameters included anesthesia modality, blood transfusion, operation duration, and duration of hospital stay. Second, the efficacy and safety parameters relevant to the postoperative results included neuralgia relief, primary and secondary success rates, complication rate, infection rate, and reintervention rate.

Primary clinical success of EI and UGTI was determined as the exclusion of the PA without residual filling on postoperative angiogram and no complications or reintervention throughout the follow‐up duration. Secondary clinical success referred to reinterventions with results matching the criteria for primary clinical success^11^, ^17^. Primary clinical success of OS was defined as resection of PA with vascular patency or ligation, no postoperative bleeding at the residual or anastomotic site, and no complications or reintervention throughout the follow‐up duration. Secondary clinical success of OS referred to reinterventions with results meeting the criteria for primary clinical success.

### Statistical analysis

All statistical analyses were carried out using SPSS (version 17.0, IBM, Chicago, IL, USA). Results are presented as mean ± standard deviation or proportions. ANOVA was used for continuous variables, such as age, diagnostic period, sac size, volume of blood transfusion, operation duration, and duration of hospital stay. Categorical variables included sex, affected side, location, donor artery, anesthesia modality, success and complication rates of the treatment options. These variables were calculated and compared with Fisher's exact test. A *p* value < 0.05 was considered statistically significant.

## Results

### Follow‐Up

The patients were followed up with a duration of 14 months (range, 2–60 months). The patients underwent UGTI were followed up with Duplex ultrasound the next day and 1 month after operation. The patients treated with EI and OS were examined with color Doppler ultrasound at 1 day, 1 month, 3 months, and 1 year after operation. All patients were followed by telephone thereafter and consulted with a doctor if necessary.

### General data

There were 18 females and 20 males, with an average age of 56.47 ± 14.08 years (range, 17–87 years). There were 15, 10, and 13 patients in the OS, EI, and UGTI groups, respectively. Among them, 10 PAs resulted from trauma and 28 were iatrogenic. Thirty PAs were located in the lower extremities and eight in the upper limbs, 19 cases on left side and 19 on another side. The patients were diagnosed with an average time of 45 days (range, 2–390 days) delayed after initial injury. Patients typically presented with a progressively enlarging and pulsatile mass, accompanied with pain in most cases. There were 25 patients with comorbidities. Demographic characteristics are shown in Table 1.

**TABLE 1:** Demographic characteristics of pseudoaneurysm patients

Variables	OS (n = 15)	EI (n = 10)	UGTI (n = 13)	Statistic values	*p*‐value
Age (years)	56.47 ± 14.08	57.70 ± 15.54	55.00 ± 19.32	*F* = 0.078	*p* > 0.05
Sex (female: male)	8:7	4:6	6:7	χ^*2*^ *=* 0.44	*p* > 0.05
Affected side (left: right)	8:7	5:5	6:7	χ^*2*^ *=* 0.144	*p* > 0.05
Causes (trauma: iatrogenic)	4:11	2:8	4:9	χ^*2*^ *=* 0.34	*p* > 0.05
Diagnostic period (days)	37.00 ± 47.95	35.58 ± 42.75	55.58 ± 119.22	F = 0.251	*p* > 0.05
Location (T: LL: UE)	8:2:5	7:2:1	10:1:2	χ^*2*^ *=* 3.13	*p* > 0.05
Donor artery (IA: EA)	11:4	7:3	11:2	χ^*2*^ *=* 0.79	*p* > 0.05
Sac size (cm^3^)	517.56 ± 927.53	287.39 ± 334.14	51.44 ± 36.48	*F* = 2.029	*p* > 0.05

*Notes*: Values are expressed as mean ± standard deviation or absolute numbers.

Abbreviations: EA, expendable artery; EI, endovascular intervention; IA, inexpendable artery; LL, lower leg; OS, open surgery; T, thigh; UE, upper extremity; UGTI, ultrasound‐guided thrombin injection.

### Procedure‐related characteristics

The comparison of results of the different procedures are shown in Table 2. UGTI required no anesthesia, whereas EI and OS were performed under anesthesia. General anesthesia was more frequently used in the OS group than in the EI group. The anesthesia modalities had a significant difference in any two groups (*χ* ^*2*^ *=* 39.80, *p* < 0.05).

**TABLE 2:** Comparison of the treatment procedures for pseudoaneurysms

Variables	OS (n = 15)	EI (n = 10)	UGTI (n = 13)	Statistic values	*p*‐value
Anesthesia (L:GA:N)	11:4:0	9:1:0	0:0:13	*χ* ^*2*^ *= 39.80*	*p* < 0.05
Blood transfusion (Units)	3.6 ± 6.0	0.8 ± 2.5	0.0 ± 0.0	*F = 3.03*	*p* < 0.05*
Operation duration (minutes)	80.0 ± 41.9	56.0 ± 8.4	22.7 ± 5.3	*F = 15.69*	*p* < 0.05
Hospital stay (days)	47.7 ± 39.0	31.5 ± 17.6	16.3 ± 9.5	*F = 47.73*	*p* < 0.05

*Notes*: Values are expressed as mean ± standard deviation or absolute numbers. *:*P‐*value between the OS and UGTI groups.

Abbreviations: EI, endovascular intervention; GA, general anesthesia; L, reginal anesthesia; N, no anesthesia; OS, open surgery; UGTI, ultrasound‐guided thrombin injection.

The patients in the OS and EI groups required blood transfusion during operation, while those in the UGTI group did not need any transfusion. The volumes of blood transfusion (Units) were 3.6 ± 6.00, 8.0 ± 2.5, and 0.0 ± 0.0 respectively. There was significant difference only between the OS and UGTI groups (*χ* ^*2*^ *=* 39.80, *p* < 0.05).

The operation durations (minutes) were 80.0 ± 41.9, 56.0 ± 8.4, and 22.7 ± 5.3 in the OS, EI, and UGTI groups, respectively, with significant differences between any two groups (*F* = 15.69, *p* < 0.05). The mean duration of OS was the longest, followed by EI, and then UGTI.

The hospital stay (days) was 47.7 ± 39.0, 31.5 ± 17.6, and 16.3 ± 9.5 in the OS, EI, and UGTI groups, respectively. The patients undergoing OS had to stay in the hospital the longest, while those treated with UGTI stayed the shortest. There were significant differences between any two groups (*F* = 47.73, *p* < 0.05).

### Efficacy

Primary clinical success was observed in 12, 9, and 11 patients in the OS, EI, and UGTI groups. The primary clinical success rates were 80%, 90%, and 92.3% in the OS, EI, and UGTI groups, respectively, with no significant difference between any two groups (*χ* ^*2*^ = 0.34, *p* > 0.05). Secondary clinical success rates were 100% for all the groups. There was no PA recurrence in any groups. Neuralgia was relieved in all patients. All of the repaired arteries, covered stents, vascular anastomoses, and vein graft were patent throughout the follow‐up period.

### Complications

A total of five patients (13.1%) had complications—three in the OS group, one in the EI group, and one in the UGTI group—with complication rates of 20%, 10%, and 7.7%, respectively. There was no significant difference between any two groups (*χ* ^*2*^ = 1.00, *p* > 0.05). Wound infection was documented in three patients postoperatively: two in the OS group and one in the EI group. The infection rates were 13.3% (2/15), 10% (1/10), and 0% (0/13) in the OS, EI, and UGTI groups, respectively, with no significant difference between any two groups (*χ* ^*2*^ = 1.80, *p* > 0.05). Among the three patients, one patient underwent further surgery, while the other two were treated with wound dressing changes and medication. Reinterventions were performed for two patients, one with autogenous vein graft who developed anastomotic breakdown and one due to UGTI failure. The reintervention rates of the OS, EI, and UGTI groups were 6.7% (1/15), 0% (0/10), and 7.7% (1/13), respectively, with no significant difference between any two groups (*χ* ^*2*^ = 0.95, *p* > 0.05). One patient treated with EI had a mild stenosis 1 year after operation. However, the patient had no signs of distal ischemia and did not require reintervention. No patient presented with ischemia of the affected extremity. There was no procedure‐related mortality in any groups.

## Discussion

Our study has shown that OS, EI, and UGTI are effective and safe treatments for PAs, with UGTI entailing no anesthesia and blood transfusion, and associated with the shortest operation time and hospital stay. To our knowledge, this is the first study that compares the outcomes among OS, EI, and UGTI even if it only represents a small case series.

### Choice of therapy

In our study, PAs caused by trauma and iatrogenic injuries can be treated with OS, EI, and UGTI. The early clinical outcomes were satisfactory. In our study, ultrasound‐guided compression therapy was not chosen due to its high failure rates, discomfort due to the long compression time required, and replaced by UGTI in most cases^2^. For asymptomatic PAs with diameter no more than 2 cm, some authors recommend intensive surveillance instead of surgery due to the possibility of spontaneous thrombosis^6^, ^8^. Symptomatic PAs or those with generally large (>2 cm) diameter may require intervention.

UGTI is utilized as the first‐line therapy for PAs. In our study, none of the patients in the UGTI group required any kind of anesthesia. All procedures were well tolerated by the patients^11^. In contrast, in the EI and OS groups, all patients received anesthesia, which may increase the complication risk of the surgical procedure. There was no need of blood transfusion for UGTI and this procedure is associated with the shortest operation time and duration of hospital stay. These findings suggest that UGTI is a simple, fast, and efficient therapy. UGTI was effective in achieving PA thrombosis. It is reported that thrombin emboli risk may increase with decreases in the sac size and its neck length, and increases in its neck width^11^. Distal embolization risk can be minimized by slow injection and avoiding aneurysms with short wide necks. In our study, one patient had PA recurrence after a successful intervention. Saydam *et al*. considered UGTI to be safer and more effective than OS for patients with iatrogenic femoral PAs^11^. However, UGTI is not appropriate for patients with imminent PA rupture, superficial skin necrosis, distal ischemia, or infection, and is contraindicated for patients with allergy to thrombin^15^, ^18^.

In addition to UGTI, EI is another novel therapy for PAs in recent years. However, its success rate varies. Based on our study, EI is a procedure with high success and low complication rates. There is only one case of infection. The donor artery remains patency of the vascular lumen 14 months after operation. The indices related to the procedure are better than those in the OS group, but inferior to those in the UGTI group, except for the amount of blood transfusion. In this regard, the amount of blood transfusion in the EI group is smaller than that in the OS group, but larger than that in the UGTI group. However, no significant difference was detected. In our treatment strategy, EI is indicated for PAs that are difficult to access percutaneously, such as PAs of the profunda femoris or the external iliac, those not suitable for OS and those failed by UGTI, which is consistent with previously documented opinion^15^, ^19^. Despite its advantages, the high incidence of lumen thromboembolism should be considered^20^. Stent grafting is optimal for arteries with relatively large diameters but unsuitable for ankle and crural PAs^21^, ^22^. Some investigators suggest that EI could be reserved as an alternative modality for selected cases^23^. Stents are not appropriate for crossing joints due to the risk of pinch off or occlusion during articular range of motion. Tortuous donor arteries and infected PAs are contraindications for EI^24^.

Compared with the two aforementioned therapies, OS represents a traditional approach. It can be applied for all PA treatment. However, this treatment method might be progressively replaced by UGTI and EI. OS is indicated for patients with PAs for which UGTI and EI have failed, those with massive hematoma and distal ischemia or signs of nerve compression, imminent rupture, infected PAs, associated arteriovenous fistula, and a location above the inguinal ligament^3^. Devendra *et al*. reported 14 cases of PAs treated with OS following orthopedic trauma. Although the result was satisfactory, the procedure required a large volume of blood transfusion and prolonged duration of hospital stay^10^.

Therefore, in light of the complexity of PAs, treatment options should be customized and carefully selected.

### Comparison of efficacy and safety

In our study, the primary clinical success rates were 80% in the OS group, 90% in the EI group, and 92.3% in the UGTI group. There was no significant difference among three groups. Secondary clinical success rates were 100% for all three groups. There were five patients with complications, with no significant difference among three groups. The results demonstrate that all three interventions are efficacious and safe for the treatment of PAs.

The OS option relies on the artery type, location, and sac size, and include end‐to‐end anastomosis, reconstruction with autogenous saphenous vein suitable for vital arteries, and ligation for expendable arteries. Devendra *et al*. reported 14 cases of PAs related to orthopedic trauma. Excision of the sac and ligation of the donor artery was performed in eight patients, while the repair of a major artery was done in six patients^10^. Yetkin *et al*. reported a series of nine patients diagnosed with brachial PAs treated with aneurysmal resection combined with saphenous vein graft interposition. OS was an efficacious method, with early and late patency rates of 100%, and relief of pain and distal ischemia^25^. For PAs at the crural level and around the foot and ankle, surgery was commonly used^13^, ^21^. Although minimally invasive interventions have been gradually favored, OS remains to be the standard treatment method for ankle PAs^26^.

Minimally invasive treatments, which included UGTI and EI (such as intraluminal coil embolization and stent grafting), have yielded promising results and become preferred treatments for PA in recent years^27^, ^28^. UGTI has replaced ultrasound‐guided compression^29^. This procedure has a high success rate of more than 90%, and low complication rate^18^. Kleczynski *et al*. reported 82 patients with iatrogenic extremity PAs treated with UGTI, with a primary success rate of 92.7%^30^. Similarly, Garvin *et al*. reported 14 patients with iatrogenic brachial and radial artery PAs treated with UGTI, with a success rate of 86%^31^. Saydam *et al*. considered UGTI to be safer and more effective than OS for PAs^11^. Conversely, EI aims at exclusion of PA and the donor artery. This method has also achieved satisfactory results^32^. Shreve *et al*. reported that EI treatment of traumatic PAs is efficacious with minimal complication rates and low reintervention requirements. In their series of 35 cases, 13 were diagnosed with extremity PAs, and only one patient underwent repeat embolization^17^. Mohan *et al*. also reported 13 patients with traumatic PAs in the extremity treated with EI, among whom 12 patients showed significant clinical improvement. EI can be applied for traumatic pseudoaneurysms in both pediatric and adult patients^28^. However, the high incidence of lumen thromboembolism has been a significant challenge to its success. Poor patency due to stent obstruction has been reported for crural PAs treated with covered stent grafting^13^, ^33^. The long‐term efficacy of EI is still unclear. Until now, there is no consensus on the treatment algorithm for PAs^8^.

The most common complications of PAs are neurologic dysfunction, distal embolization, and rupture^23^, ^34^, ^35^. In our series, there were five patients (13.1%) who developed complications: three wound infections and two reinterventions. Among the three patients with wound infections, only one underwent further surgery, while the rest were managed conservatively. Reintervention was performed for a patient who underwent autogenous vein grafting and developed anastomotic breakdown, and for another patient with failed UGTI. There was no recurrence of PA, distal ischemia, vascular occlusion, and procedure‐related mortality in our study cohort.

### Suggestions of treatment strategy

Although the traumatic and iatrogenic PAs can be treated with all the three aforementioned methods, there remains no consensus on the practice guidelines for the management due to the high variability of presentation and the limited availability of relevant high‐quality literature. To date, most opinions arise from case series in literature. The treatment strategy depends on several factors, such as anatomic location, neck width, sac size, features of the donor artery, rupture risk, patient comorbidity, and physician's decision. Because of their unpredictable nature, surgical treatment is warranted for asymptomatic PAs with or without spontaneous thrombosis^2^. For patients with imminent or ruptured PAs, neuralgia caused by nerve compression, and severe distal ischemia, surgery should be performed urgently^3^, ^4^. Saydam *et al*. reported their treatment protocol for femoral artery PAs. They presented their experience with patients who had iatrogenic PAs treated with UGTI and OS. They recommended UGTI as the first‐line option for PAs^11^. Shah *et al*.^36^ and Henry *et al*.^2^ also provided their treatment strategies for PAs. However, the patients enrolled in these studies were all iatrogenic PAs. Regarding the major causes of PAs, it is important to include those arising from trauma. Based on our experiences and literature review, UGTI represents an optimal treatment for PA with improved effectiveness and easy performance in comparison to the other two methods. We considered our workflow pragmatic, which warranted recommendation as a guideline for treating PAs.

### Limitations

This study has several limitations. First, this is a retrospective analysis and the treatment options are not randomized. Second, the follow‐up is relatively short, and long‐term outcomes should be evaluated in future studies. Finally, the size of the study cohort is relatively small.

### Conclusion

Traumatic and iatrogenic PAs remain a challenging clinical problem. OS, EI, and UGTI are efficacious and safe options for PA management. The correct treatment should be based on a thorough understanding of PA pattern and its characteristics. UGTI is simpler, faster, and more effective than the other two methods. EI and OS may be reserved for those with failed UGTI. OS can be utilized as a final solution. Satisfactory clinical outcomes can be achieved when PAs are correctly approached. We recommend a considerably pragmatic workflow for treatment.

## DISCLOSURE

The authors have no conflicts of interest to declare.
